# Inhibition of proliferation of rabbit lens epithelial cells by S-phase kinase-interacting protein 2 targeting small interfering RNA

**Published:** 2010-05-25

**Authors:** Ying Su, Feng Wang, Qinghui Yan, Yan Teng, Hao Cui

**Affiliations:** Department of Ophthalmology, First Clinic College of Harbin Medical University, Harbin, China

## Abstract

**Purpose:**

Improper proliferation of lens epithelial cells is causally related to posterior capsule opacification. In the present study, we investigated whether small interfering RNA (siRNA)-mediated gene silencing of S-phase kinase-interacting protein 2 (Skp2) can be employed to inhibit rabbit lens epithelial cell (rLEC) proliferation by increasing the p27^kip1^ level.

**Methods:**

A plasmid containing *Skp2* siRNA was used to decrease the high constitutive level of Skp2 protein in rLECs, which can lead to consequent degradation of p27^kip1^. Protein expression of Skp2 and p27^kip1^ was detected by immunocytochemistry and western blot. Cell viability was measured using the tetrazolium reduction (3-(4,5-dimethylthiazolyl-2-)-2,5-diphenyltetrazoliumbromide [MTT]) assay. Cell proliferation was assayed by cell counts, immunocytochemistry, and western blot by using antibodies against proliferating cell nuclear antigen.

**Results:**

Immunocytochemistry and western blot showed a decreased level of Skp2 and increased level of p27^kip1^ in cells transfected with *pSkp2* siRNA but not in vehicle transfection and uninfected cells. MTT assay showed that cell viability significantly declined in rLECs transfected with *Skp2* siRNA. *Skp2* siRNA transfected cells showed significantly less 59-bromodeoxyuridine- and proliferating cell nuclear antigen-positive staining compared with control cells.

**Conclusions:**

*Skp2* siRNA inhibits cell proliferation and decreases cell viability of rLECs in vitro by suppression of p27^kip1^ downregulation. Our findings suggest that siRNA-mediated gene silencing of *Skp2* can be a novel gene therapy for posterior capsule opacification induced by LEC abnormal proliferation.

## Introduction

Lens epithelial cells (LECs) at the equatorial region continue proliferating and differentiating throughout life. Both withdrawal from the cell cycle and differentiation into fibers is essential for lens formation and lens transparency [1]. Improper proliferation of lens epithelial cells after cataract surgery is causally related to posterior capsule opacification (PCO) [2,3]. However, the mechanisms underlying lens cell proliferation are still unclear.

S-phase kinase-interacting protein 2 (Skp2) has been identified as an E3 ubiquitin ligase [4] that targets p27^kip1^ for ubiquitination [5] and plays an important role in cell-cycle regulation [6]. Skp-cullin-F-box complexes represent an evolutionarily conserved class of E3 enzymes containing four subunits: Skp1, Cul1, F box proteins, and Roc1/Rbx1 [7]. Skp2 is a rate-limiting component of the machinery that is specifically required for p27^kip1^ ubiquitination and degradation [5]. Skp2 is frequently overexpressed in tumor cell lines, and forced expression of Skp2 in quiescent fibroblasts induces DNA synthesis [8]. Yoshida et al. [9] showed that expression of Skp2 can be detected in lens epithelial cells. However, the role of Skp2 in lens epithelial cell proliferation is still uncertain.

RNA interference (RNAi) can easily and effectively inhibit the expression of a specific gene [10]. The RNAi process is mediated through small, double-stranded RNA molecules called small interfering RNAs (siRNAs), which specifically trigger the cleavage and subsequent degradation of their target mRNA in a sequence-dependent manner. Therefore, RNAi can prevent synthesis of a protein encoded by the target mRNA [11]. Recently, RNAi-mediated gene silencing has been shown to be efficient in mammalian cells, and this has led to the increasing feasibility of RNAi technology for the therapy of certain human diseases [12]. IkkapaB kinase subunit beta (IKKβ) targeting siRNA was reported to inhibit the proliferation of in vitro human Tenon’s capsule fibroblast [13]. Our recent study showed that transfection of *Skp2* siRNA can effectively inhibit the proliferation of rabbit tenon’s fibroblast cells after glaucoma surgery [14].

In this study, we examined the expression of Skp2 in rabbit LEC (rLEC) and investigated if siRNA-mediated gene silencing of Skp2 could inhibit p27^kip1^ downregulation and repress rLEC proliferation in vitro.

## Methods

All experimental procedures were carried out in accordance with Harbin Medical University guidelines for animal care and the Guide for Care and Use of Laboratory Animals published by the US National Institutes of Health (NIH publication number 85–23, revised 1996).

### Source of reagents

Keratin antibody, skp2 antibody, and streptavidin biotin complex (SABC) kit were purchased from Boster company (Wuhan, China). P27 kip1 antibody, PCNA antibody and Enhanced chemiluminescence kit were from Santa cruz biotechnology Inc (Santa Cruz, CA). Fluorescein-conjugated anti-sheep IgG was purchased from Zhongshan biotechnology (Beijing, China). Vectashield mounting medium was from Vector laboratories (Burlington, Canada). Modified Eagle's medium (MEM) was from Gibco (Burlington, VT). Fetal calf serum, Trizol TM were from Invitrogen (Carlsbad, CA). Hanks solution was from Hyclone (Logan, UT). Poly-lysine and Phosphate buffer solution (PBS) were purchased from Sigma (St. Louis, MO). Culture plate was from BD Biosciences (San Jose, CA). Hiperfect transfection reagent was purchased from Qiagen gene company (Hilden, Germany). Hybond-P polyvinylidene difluoride (PVDF) membrane was from Amersham Pharmacia Biotech (Piscataway, NJ).  3-(4,5-dimethylthiazolyl-2-)-2,5-diphenyltetrazoliumbromide (MTT) was from Sigma. MTT cell proliferation kit was purchased from ATCC (Manassas, VA). Metafectene Proreagent was purchased from Biontex, company (Martinstried, Germany). G418 was purchased from Life Technologies company (Carlsbad, CA). Anti- GAPDH antibody was purchased from Abcam company (Cambridge, MA). Mouse anti-BrdU-fluorescein primary antibody was purchased from Roche (Madison,WI).

### Equipment used

Fluorescence microscope (IX70) and optic microscope was purchased from Olympus company (Tokyo, Japan). CO_2_ incubator (BB16HF) and ltraclean work table (D8C-010) were purchased from Heal Force (Hong Kong,China). Incubation plate was from Coster corporation (Cambridge, MA). Cell Counter (Coulter Z1) was purchased from Coulter company (Hialeah, FL).

### Cell culture of rabbit lens epithelial cells

Adult albino rabbits weighing between 2 and 3 kg, purchased from experimental animal center of Harbin Medical University, were used for experiment. The rabbits used in this investigation were handled in accordance with the tenets of the Association for Research in Vision and Ophthalmology (ARVO) Statement for the Use of Animals in Ophthalmic and Vision Research. Twelve-week-old rabbits weighing 1–1.5 kg were killed by CO_2_ inhalation. The entire eye was removed and dipped in 75% ethanol for 30 s and was then opened from the posterior segment. The dissection procedure was performed with sterile instruments under a laminar flow hood. Lenses were dissected carefully using a posterior approach and were washed three times in PBS to remove attached pigments and vitreous. The capsule epithelium was dissected by fine forceps and placed in a 35-mm^2^ culture dish where it adhered to the plastic. Some drops of culture medium were applied to the epithelium specimens to prevent drying, and the dishes were placed in a humidified CO_2_ incubator (5% CO_2_, 37 °C) for 6 h to allow firm attachment of the capsules. Another 2 ml of culture medium was then added, and the capsules were left for 3 or 4 days before the first trypsinization and subculturing. The medium, which was changed every 2 days, was modified Eagle's medium (MEM) with 10% fetal bovine serum (FBS), supplemented with 2 mM L-glutamine, 100 U/ml penicillin, and 100 μg/ml streptomycin (all from Sigma).

rLECs were cultured in a six-well plate with a sterile coverslip. All culture media were as described for the epithelium specimens. Cells used in subsequent experiments were generally from passages two to three. All cells were grown at 37 °C with 5% CO_2_ ventilation.

### Plasmids and transfection

Vector pSuppressorNeo generates biologically active siRNAs from the U6promoter. Synthetic oligonucleotide primers (5′-TCG AGG GAG UGA CAA AGA CUU UGG AGU ACU GCA AAG UCU UUG UCA CUC CCU UUU U-3′) containing XhoI and XbaI overhangs were annealed and then were introduced into pSuppressor Neovector. Oligonucleotide sequences correspond to a 19-nucleotide sequence from Skp2 (nucleotides 114–133), which is separated by an eight-nucleotide linker from the reverse complement of the same 19-nucleotide sequence. A transcriptional termination (UUUUU) was added to the end of the oligonucleotide. We used a circular control plasmid, which contains a scrambled sequence, as a control. rLECs transfected with pSuppressor containing Skp2 siRNA, pSuppressor only, and medium served as the experimental group, vehicle control group, and blank control group, respectively. Transfection was performed in 60-mm plates, using 2 µg (1 µg/µl) of vector in 10 µl of Metafectene Proreagent. After 48 h of transfection, cells were treated with G418 for 2 weeks for positive clone selection. After G418 treatment, we cloned several stable transfectant cells. Each clone was screened for expression of Skp2 by western blot analysis.

### Immunocytochemistry

Cells cultured on coverslips were fixed with 4% paraformaldehyde for 10 min at room temperature, permeabilized, and blocked with 0.1% Triton X-100 and 5% goat serum for 30 min. Cells were incubated with rabbit anti-keratin antibody (1:500) at 4 °C overnight, followed by goat-antirabbit IgG (1:500) for 1 h at room temperature. Cells were then incubated with SABC at 37 °C for 30 min and colored with diaminobenzidine (DAB). This was followed by dehydration and dimethyl benzene treatment, and then slides were mounted. Controls were stained by omitting the primary antibody. Skp2 monoclonal antibody (1:500 dilution), p27^kip1^ antibody (1:500 dilution), and PCNA antibody (1:1,000 dilution) were also used for immunofluorescence staining experiments.

### Western blot analysis

The protein expression of Skp2, p27^kip1^, and PCNA from three different rLEC samples in each treatment was examined by western blot analysis. rLEC cells (6×10^5^)  treated with pSuppressor containing Skp2 siRNA, pSuppressor only, and medium were prepared in extraction buffer containing 50 mM Tris-HCl (pH 7.4), 150 mM NaCl, 1% Triton X-100, 0.1% sodium dodecyl sulfate (SDS), 1 mM EDTA, 1 mM 4-(2-Aminoethyl)-benzenesulfonyl (AEBSF), 20 μg/ml aprotinin, and 20 μg/ml leupeptin. Equal amounts of total protein (10 μg) were separated by 10% sodium dodecyl sulfate polyacrylamide gel electrophoresis (SDS-PAGE) and transferred to a Hybond-P polyvinylidene difluoride membrane. After blocking with 5% nonfat dry milk in PBS with 0.1% Tween-20, membranes were probed with anti-Skp2 (1:500), anti-p27^kip1^ (1:500), or anti-PCNA mouse monoclonal antibodies (1:1,000), followed by incubation with the appropriate secondary antibody. Visualization of the protein bands was performed by the enhanced chemiluminescence kit. A parallel western blot was probed with an anti-GAPDH antibody as a loading control. Band intensity was quantified using Quantity One 4.4.1 software (Bio-Rad, Hercules, CA).

### 3-(4,5-dimethylthiazolyl-2-)-2,5-diphenyltetrazoliumbromide assay

Cell viability was examined by the MTT cell proliferation kit following the instructions of the manufacturer. The assay is based on measuring the reduction of yellow tetrazolium to purple formazan as facilitated by dehydrogenases of metabolically active cells. The intracellular formazan can be solubilized and quantified by spectrophotometric means. Quadruple samples of rLEC were grown on 96-well plates and were infected with 2 µg (1 µg/µl) of either of the two vectors in 10 µl of Metafectene proreagent or were not infected. After 2, 4, 6, 8, and 14 days, wells were incubated in a medium containing yellow tetrazolium for 20 h.

### Cell proliferation and bromodeoxyuridine incorporation

Cells (5.0×10^3^) were plated onto a 24-well multiwell plate (Falcon; Becton Dickinson, Franklin Lakes, NJ) and allowed to attach for 24 h. The culture medium was then replaced with fresh medium. Cells were trypsinized and counted with a cell counter at 0, 2, 4, and 6 days. For bromodeoxyuridine (Brdu) incorporation, cells growing on coverslips were incubated with 10 µm/l Brdu (Sigma) for 3 h. After fixing in cold methanol/acetone 1:1 for 10 min, the cells were sequentially incubated in 1.5 mol/l HCl for 10 min. Cells were then washed with PBS and incubated with mouse anti-BrdUrd-fluorescein primary antibody for 1 h. The cells were washed four times with PBS. The nuclei were simultaneously stained with 10 µg/ml of 4V, 6-diamidino-2-phenylindole. Cells with different BrdUrd incorporation patterns were analyzed and counted with a conventional fluorescence microscope.

### Statistical analysis

The data were analyzed by the two-tailed Student *t* test using SPSS 10.0; p<0.05 was considered significant.

## Results

### The identification of rabbit lens epithelial cells

**Figure 1:**
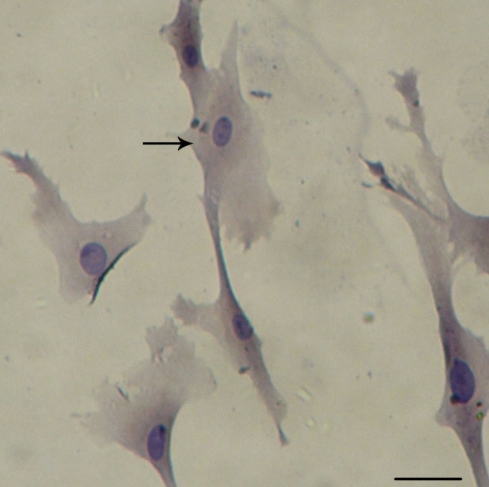
Identification of rabbit lens epithelial cells in vitro. Immunocytochemistry demonstrated expression of keratin protein in the nucleus of cultured cells, indicating that they were rLECs. The arrow indicates one rLEC. Scale bar is equal to 80 μm.

An immunocytochemistry assay of keratin, a special cell marker of rLECs, was used in our study to identify rLECs. As shown in Figure 1, cultured cells expressed keratin protein (brown staining) in the nucleus, which indicated rLECs.

### Downregulation of Skp2 protein by small interfering ribonucleic acid in rabbit lens epithelial cells

**Figure 2:**
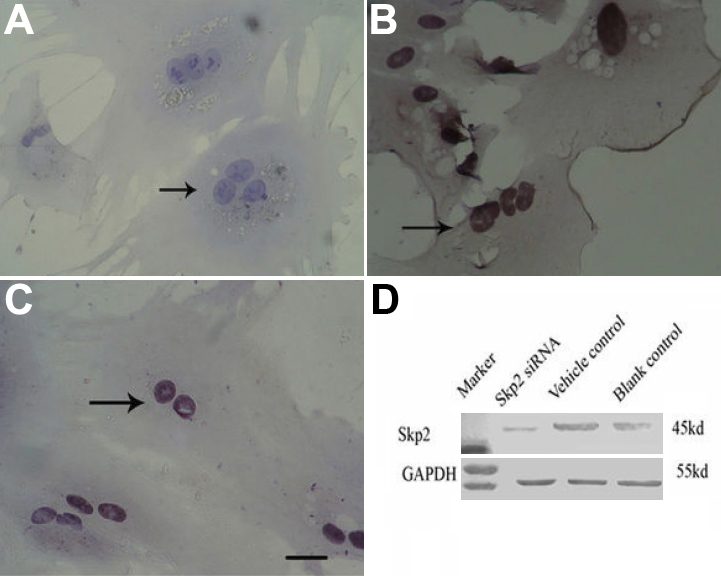
Downregulation of S phase-kinase-interacting protein 2 protein by small interfering ribonucleic acid in rabbit len epithelial cells in vitro. Immunocytochemistry staining (one representative of three experiments) indicated high constitutive levels of Skp2 protein (indicated by arrow) in the nucleolus of rLECs transfected with pSuppressor vehicle (**B**) or without transfection (**C**). Transfection with *Skp2* siRNA dramatically decreased the expression of Skp2 protein in rLECs (**A**). Scale bar is equal to 40 μm. After 48 h of transfection and 2 weeks of treatment with G418, several stable transfectant cells were cloned. Western blot analysis (one representative of three experiments) showed that high constitutive Skp2 expression can be detected in rLECs of the vehicle control group and blank control group (**D**). However, little expression of Skp2 was detected in the experimental group, indicating transfection with *Skp2* siRNA can inhibit expression of Skp2 in rLECs in vitro (**D**). GAPDH served as loading control.

After 48 h of transfection and 2 weeks of treatment with G418, we cloned several stable transfectant cells. Immunofluorescence staining demonstrated high constitutive expression of Skp2 protein in the nucleolus of rLECs transfected with pSuppressor vehicle (Figure 2B) or without transfection (Figure 2C). Transfection with *Skp2* siRNA dramatically decreased the expression of Skp2 protein in cells of rLEC (Figure 2A). Each clone was also screened for expression of Skp2 by western blot analysis. Skp2 expression can be detected in rLECs of the vehicle control group and the blank control group (Figure 2D, lanes 2 and 3, respectively). However, there was little expression of Skp2 in the experimental group, which indicated that transfection with *Skp2* siRNA can inhibit expression of Skp2 in rLECs in vitro (Figure 2D, lane 1).

### Upregulation of p27^kip1^ protein by Skp2 small interfering ribonucleic acid in rabbit lens epithelial cells

**Figure 3:**
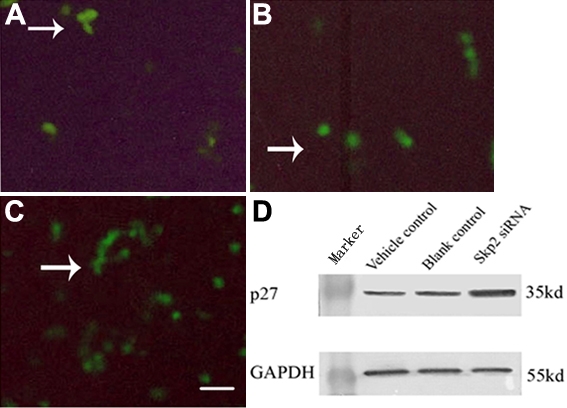
S phase-kinase-interacting protein 2 protein by small interfering ribonucleic acid induced p27 kinase inhibition protein 1in rabbit lens epithelial cells in vitro. Immunofluorescence staining indicated the expression of p27^kip1^ dramatically increased in rLECs transfected with *Skp2* siRNA (**C**) when compared with rLECs transfected with pSuppressor vehicle (**A**) or without transfection (**B**). The scale bar is equal to 40 μm. Western blot (one representative of three experiments) showed that the expression of p27^kip1^ dramatically increased in rLECs transfected with *Skp2* siRNA (**D**) when compared with rLECs transfected with pSuppressor vehicle (**D**) or without transfection (**D**). GAPDH served as the loading control.

Skp2 is required for the ubiquitination and consequent degradation of p27^kip1^ [2]. Immunofluorescence staining demonstrated little expression of p27 protein in the rLECs transfected with pSuppressor vehicle (Figure 3A) or without transfection (Figure 3B). Transfection with *Skp2* siRNA increased the expression of p27^kip1^ protein in rLECs (Figure 3C) in vitro. Western blot analysis in our study demonstrated that p27^kip1^ expression of rLECs in the experimental group increased (Figure 3D, lane 3) when expression of Skp2 decreased (Figure 2). Upregulation of p27^kip1^ made it possible for us to investigate the effect of p27^kip1^ inhibition on the proliferation of rLECs.

### Skp2 small interfering ribonucleic acid decreased the rabbit lens epithelial cells viability and proliferation in vitro

#### 3-(4,5-dimethylthiazolyl-2-)-2,5-diphenyltetrazoliumbromide (MTT)

**Figure 4:**
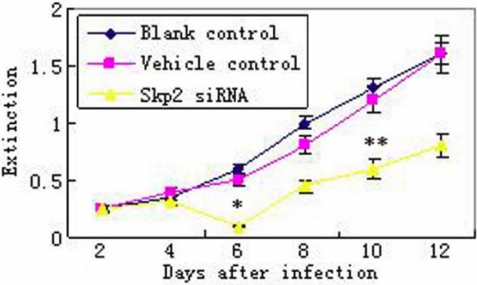
Cell viability assay by 3-(4,5-dimethylthiazolyl-2-)-2,5-diphenyltetrazoliumbromide (MTT). After transfection with *Skp2* siRNA, cell viability was significantly decreased in cultured rLECs at days 6 and 10 compared with the vehicle control and blank control. The asterisk indicates p<0.05 versus the vehicle and blank controls, while the double asterisk indicates p<0.01 versus the vehicle and blank controls (n=32).

As shown in Figure 4, cell viability did not show any significant differences after vehicle transfection compared with the blank control group, indicating that the plasmid did not influence cell metabolism. However, cell viability in *Skp2* siRNA transfected rLECs (experimental group) significantly decreased at 6 and 10 days after transfection when compared with the vehicle control and blank control.

#### Incorporation of bromodeoxyuridine

**Figure 5:**
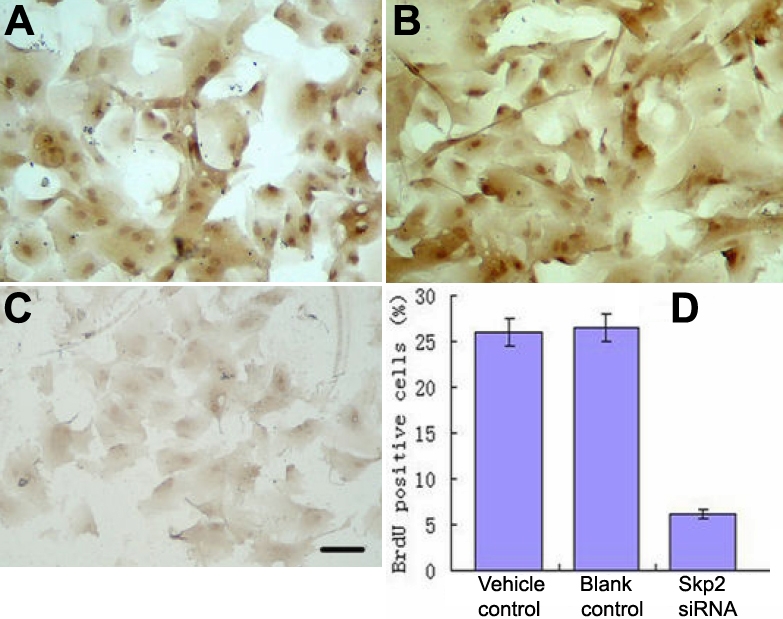
S phase-kinase-interacting protein 2 protein by small interfering ribonucleic acid inhibited cell proliferation of rabbit len epithelial cells in vitro (3-(4,5-dimethylthiazolyl-2-)-2,5-diphenyltetrazoliumbromide). For Brdu incorporation, cells growing on coverslips were incubated with Brdu. Incorporated Brdu was detected with antibodies as described in the Materials and Methods. High levels of BrdU with dense brown staining can be detected in rLEC of vehicle control (**A**) and blank control (**B**), but decreased in Skp2 siRNA transfectant cells (**C**). Statistical analysis (**D**) after cell counting showed that Brdu positive cells decreased after rLEC transfection with Skp2 siRNA compared with control cells. p<0.01 versus vehicle and blank control (n=32).

High-level staining of Brdu was detected in the rLECs of the vehicle control (Figure 5A) and blank control (Figure 5B) but dramatically decreased in *Skp2* siRNA transfectant cells (Figure 5C). Statistical analysis (Figure 5D) after cell counting showed that BrdU-positive cells in rLECs transfected with *Skp2* siRNA significantly decreased compared with control cells (p<0.01 versus vehicle and blank controls).

### PCNA protein expression in rLECs after transfection with *Skp2* siRNA

**Figure 6:**
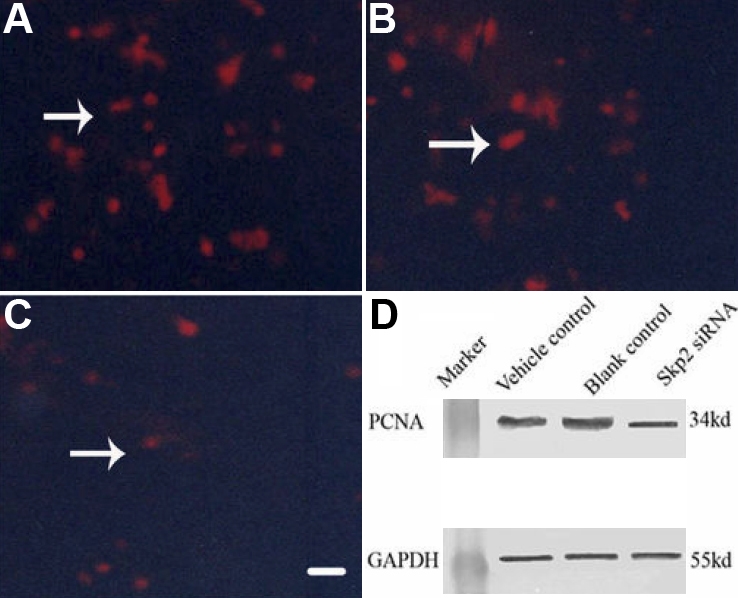
Down-regulation of proliferating cell nuclear antigen protein by mall interfering ribonucleic acid in rabbit lens epithelial cells in vitro. Immunofluorescence staining indicated the expression of PCNA decreased in rLECs transfected with *Skp2* siRNA (**C**) when compared with rLECs transfected with pSuppressor vehicle (**A**) or without transfection (**B**). The scale bar is 40 μm. Western blot showed that the expression of PCNA dramatically decreased after rLEC transfection with *Skp2* siRNA (**D**) when compared with rLEC transfection with the pSuppressor vehicle or without transfection. GAPDH served as the loading control.

PCNA is a marker that indicates the proliferation potential of cells. Immunofluorescence staining demonstrated obvious expression of PCNA protein in the rLECs transfected with the pSuppressor vehicle (Figure 6A) or without transfection (Figure 6B) in vitro. Transfection with *Skp2* siRNA decreased the expression of PCNA protein in rLECs (Figure 6C) in vitro. Western blot further confirmed that the expression of PCNA dramatically decreased after rLEC transfection with *Skp2* siRNA (Figure 6D, lane 3) when compared with rLEC transfection with pSuppressor vehicle (Figure 6D, lane 1) or without transfection (Figure 6D, lane 2).

## Discussion

Cataract surgery can induce a wound-healing response in the lens that leads to PCO. PCO incidence is approximately 50% in adults and 100% in children after cataract surgery [15] and is the most common postoperative complication of cataract surgery that causes visual loss [16]. PCO arises from residual LECs at the equator and under the anterior lens capsule after cataract surgery. These cells proliferate and migrate onto the posterior capsule underlying the intraocular lens and into the light path. Many of these cells undergo epithelial-to-mesenchymal transition (EMT), resulting in the formation of fibroblasts and spindle-like myofibroblasts, which lead to capsular opacity [16]. Clinically, there are two morphological types of PCO: fibrosis type and pearl type. The fibrosis-type PCO is caused by the proliferation and migration of LECs, which undergo EMT [15-18]. In the pearl-type PCO, LECs located at the equatorial lens region can cause regeneration of crystallin-expressing lenticular fibers and form Elschnig pearls and a Soemmering ring.

The histological features of PCO are now well established, but to date the molecular mechanisms influencing leftover LEC behavior after cataract surgery are not completely clear. LECs left behind in the capsular bag after any type of extracapsular cataract surgery are mainly responsible for PCO development [1], which involves LEC abnormal proliferation, migration, EMT, collagen deposition, and lens fiber regeneration [19-21].

Studies show that levels of several cytokines and growth factors increase in the aqueous humor and influence the behavior of the remaining LECs after cataract surgery. These factors include transforming growth factor [22-24], fibroblast growth factor 2, hepatocyte growth factor, and interleukins 1 and 6 [25,26].

Cellular proliferation is regulated primarily by cell-cycle regulation. Cell-cycle progression is regulated by a combination of positive and negative regulators. Cyclin-dependent kinase (CDKs) inhibitors (CKI) negatively regulate progression of the cell cycle by inhibiting the activity of cyclin-CDK complexes. p27^kip1^, a member of the CKI family, plays a pivotal role in the control of cell proliferation [27-29]. The level of p27^kip1^ is high during the G_0_ phase but decreases rapidly upon re-entry of the cells into the G_1_ phase. Rapid removal of p27^kip1^ at the G_0_/G_1_ transition is required for effective progression of the cell cycle to S phase [30-32]. In previous studies, Yoshida et al. [33] suggested that the disappearance of p27^kip1^ was correlated with cell proliferation in the corneal epithelium after injury. According to our study, a low level of p27^kip1^ expression was correlated with high proliferative and migratory capacity, whereas nuclear accumulation of the CKI was associated with a quiescent and static phenotype.

The level of p27^kip1^ is regulated by Skp2. Skp2 is specifically required for p27^kip1^ ubiquitination and is a rate-limiting component of the machinery that degrades phosphorylated p27^kip1^ [22]. Skp2 is constitutively expressed in normal skin tissue and scar tissue. High expression of Skp2 and decreased expression of p27^kip1^ are observed in fibroblast from pathological scar tissue [30], which indicates a negative correlation between expression of Skp2 and p27^kip1^ in fibroblasts from pathological scar tissue. However, there have been no previous studies on the role of Skp2 in LEC proliferation. Our study demonstrated that Skp2 was highly expressed and p27^kip1^ displayed little expression in rLECs. Furthermore, we showed that inhibition of Skp2 expression by siRNA enhanced the p27^kip1^ protein level and prevented rLEC proliferation.

At present, the only effective treatment of PCO is neodymium-doped yttrium aluminium garnet (Nd:YAG) laser capsulotomy, which involves clearing the visual axis by creating a central opening in the opacified posterior capsule. Although this procedure is easy and rapid, there are complications, including retinal detachment, damage to the intraocular lens (IOL), cystoid macular edema, an increase in intraocular pressure, iris hemorrhage, corneal edema, IOL subluxation, and exacerbation of localized endophthalmitis. Changes induced by Nd:YAG capsulotomy have been shown to be affected by IOL material and design. In addition, this treatment represents a considerable cost burden to national health care systems, and such laser treatment is not readily available in developing countries. Therefore, a better understanding of the pathogenic mechanism of PCO is highly desirable as a basis for improving the outcome of cataract surgery and eradicating PCO [34-36].

Approaches to inhibiting LEC proliferation after cataract surgery by gene transfer have been reported by different research groups. Malecaze et al. [37] and Counderec et al. [38] delivered adenoviral vector-mediated transfer systems, including herpes simplex virus-thymidine kinase/ganciclovir (HSV-*tk*/GCV) into rabbit LECs in vitro, which can inhibit the proliferation of rLECs. Malecaze et al. [39] demonstrated that adenoviral-mediated Bax or procaspase-3 overexpression is capable of inducing therapeutic programmed cell death in vitro and in vivo in residual lens cells and preventing PCO in a rabbit model. Kampmeier et al. [40] showed that transfection of antisense cyclin G1 retroviral vectors can inhibit proliferation of LECs. In this study we showed that siRNA targeting Skp2 prevented rLEC proliferation in vitro. Thus, the method might be used to prevent PCO after cataract surgery.

Finally, in view of the growing interest in Skp2 and p27^kip1^ as a target for drug development in inhibiting LEC proliferation following cataract surgery, it is our hope that the data presented here will help to decrease the PCO incidence from cataract surgery.

## References

[1] Liu Q Shang F Guo W Hobbs M Valverde P Reddy V Taylor A Regulation of the ubiquitin proteasome pathway in human lens epithelial cells during the cell cycle. Exp Eye Res 2004 78 197 205 14729352 10.1016/j.exer.2003.11.009

[2] Marcantonio JM Rakic JM Vrensen GF Duncan G Lens cell populations studied in human donor capsular bags with implanted intraocular lenses. Invest Ophthalmol Vis Sci 2000 41 1130 41 10752951

[3] Ursell PG Spalton DJ Pande MV Hollick EJ Barman S Boyce J Tilling K Relationship between intraocular lens biomaterials and posterior capsule opacification. J Cataract Refract Surg 1998 24 352 60 9559471 10.1016/s0886-3350(98)80323-4

[4] Ganoth D Bornstein G Ko TK The cell cycle regulatory protein Ckst is required for SCF (Skp2) mediated ubiquitinylation of P27. Nat Cell Biol 2001 3 321 4 11231585 10.1038/35060126

[5] Akimoto M Miyahara T Arai J A new delivery system for 5-fluorouracil using prodrug and converting enzyme. Br J Ophthalmol 2002 86 581 6 11973258 10.1136/bjo.86.5.581 PMC1771128

[6] Johnson KTM Rodicker F Heise K Adenoviral p53 gene transfer inhibits human Tenon’s capsule fibroblast proliferation. Br J Ophthalmol 2005 89 508 12 15774934 10.1136/bjo.2004.051664 PMC1772611

[7] Carrano AC Eytan E Hershko A Pagano M SKP2 is required for ubiquitin-mediated degradation of the CDK inhibitor p27. Nat Cell Biol 1999 1 193 9 10559916 10.1038/12013

[8] Sutterluty H Chatelain E Marti A p45SKP2 promotes p27 degradation and induces S phase in quiescent cells. Nat Cell Biol 1999 1 207 14 10559918 10.1038/12027

[9] Yoshida K Harada T Harada C Kase S Ikeda H Sakai M Nishi S Imaki J Nakayama K Nagahama H Nakayama KI Ohno S Proliferative regulation in the cornea and lens. Nippon Ganka Gakkai Zasshi 2003 107 678 86 14661541

[10] Plasterk RH RNA silencing: the genome's immune system. Science 2002 296 1263 5 12016302 10.1126/science.1072148

[11] Hannon GJ RNA interference. Nature 2002 418 244 51 12110901 10.1038/418244a

[12] Elbashir SM Harborth J Lendeckel W Duplexes of 21-nucleotide RNAs mediate RNA interference in cultured mammalian cells. Nature 2001 411 494 8 11373684 10.1038/35078107

[13] Duan Y Guan X Ge J Cationic nano-copolymers mediated IKKbeta targeting siRNA inhibit the proliferation of human Tenon's capsule fibroblasts in vitro. Mol Vis 2008 14 2616 28 19137061 PMC2613073

[14] Wang F Qi L Su Y Teng Y Cui H S phase kinase-interacting protein 2 (Skip2) targeting Small interfering RNA inhibits cell proliferation of tenon’s capsule fibroblast by increasing p27 protein level. Invest Ophthalmol Vis Sci 2010 51 1475 82 19875654 10.1167/iovs.09-4363

[15] Liu J Hales AM Chamberlain CG McAvoy JW Induction of cataract-like changes in rat lens epithelial explants by transforming growth factor beta. Invest Ophthalmol Vis Sci 1994 35 388 401 8112986

[16] Hales AM Chamberlain CG McAvoy JW Cataract induction in lenses cultured with transforming growth factor-beta. Invest Ophthalmol Vis Sci 1995 36 1709 13 7601651

[17] Saika S Okada Y Miyamoto T Ohnishi Y Ooshima A McAvoy JW Smad translocation and growth suppression in lens epithelial cells by endogenous TGF beta2 during wound repair. Exp Eye Res 2001 72 679 86 11384156 10.1006/exer.2001.1002

[18] Saika S Miyamoto T Ishida I TGF beta-Smad signalling in postoperative human lens epithelial cells. Br J Ophthalmol 2002 86 1428 33 12446380 10.1136/bjo.86.12.1428 PMC1771405

[19] Caberg JH Gilles C Berx G Savagner P Boniver J Delvenne P Transforming growth factor-β1-mediated slug and snail transcription factor up-regulation reduces the density of Langerhans cells in epithelial metaplasia by affecting E-cadherin expression. Am J Pathol 2008 172 1391 402 18385519 10.2353/ajpath.2008.071004 PMC2329847

[20] Awasthi N Guo S Wagner BJ Posterior capsular opacification: A problem reduced but not yet eradicated. Arch Ophthalmol 2009 127 555 62 19365040 10.1001/archophthalmol.2009.3

[21] Awasthi N Wang-Su ST Wagner BJ Downregulation of MMP-2 and -9 by proteasome inhibition: A possible mechanism to decrease LEC migration and prevent posterior capsular opacification. Invest Ophthalmol Vis Sci 2008 49 1998 2003 18436832 10.1167/iovs.07-0624 PMC2532066

[22] Gotoh N Perdue NR Matsushima H Sage EH Yan Q Clark JI An in vitro model of posterior capsular opacity: SPARC and TGF-β2 minimize epithelial-to-mesenchymal transition in lens epithelium. Invest Ophthalmol Vis Sci 2007 48 4679 87 17898292 10.1167/iovs.07-0091

[23] Choi J Park SY Joo C-K Transforming growth factor-1 represses E-cadherin production via slug expression in lens epithelial cells. Invest Ophthalmol Vis Sci 2007 48 2708 18 17525203 10.1167/iovs.06-0639

[24] Dawes LJ Sleeman MA Anderson IK Reddan JR Wormstone IM TGFβ/Smad4-dependent and -independent regulation of human lens epithelial cells. Invest Ophthalmol Vis Sci 2009 50 5318 27 19516008 10.1167/iovs.08-3223

[25] Meacock WR Spalton DJ Stanford MR Role of cytokines in the pathogenesis of posterior capsule opacification. Br J Ophthalmol 2000 84 332 6 10684849 10.1136/bjo.84.3.332 PMC1723397

[26] Wallentin N Wickström K Lundberg C Effect of cataract surgery on aqueous TGF- B and lens epithelial cell proliferation. Invest Ophthalmol Vis Sci 1998 39 1410 8 9660489

[27] Nelsen CJ Hansen LK Rickheim DG Chen C Stanley MW Krek W Albrecht JH Induction of hepatocyte proliferation and liver hyperplasia by the targeted expression of cyclin E and skp2. Oncogene 2001 20 1825 31 11313930 10.1038/sj.onc.1204248

[28] Nakayama K Nagahama H Minamishima Y Matsumoto M Nakamichi I Kitagawa K Shirane M Tsunematsu R Tsukiyama T Ishida N Kitagawa M Nakayama K Hatakeyama S Targeted disruption of Skp2 results in accumulation of cyclinE and p27, polyploidy and centrosome over duplication. EMBO J 2000 19 2069 81 10790373 10.1093/emboj/19.9.2069 PMC305685

[29] Wei W Ayad NG Wan Y Zhang GJ Kirschner MW Kaelin WG Jr Degradation of the SCF component Skp2 in cell-cycle phase G1 by the anaphase-promoting complex. Nature 2004 428 194 8 15014503 10.1038/nature02381

[30] Ezoe S Matsumura I Nakata S Gale K Ishihara K Minegishi N Machii T Kitamura T Yamamoto M Enver T Kanakura Y GATA2 estrogen receptor chimer are gulates cytokine dependent growth of hematopoietic cells through accumulation of p21 (WAF1) and p27 (Kip1) proteins. Blood 2002 100 3512 20 12393444 10.1182/blood-2002-04-1177

[31] Fero ML Randel E Gurley KE Roberts JM Kemp CJ The murine gene P27kip1 is haplo insufficient for tumour suppression. Nature 1998 396 177 80 9823898 10.1038/24179 PMC5395202

[32] Kudo Y Takata T Ogawa I p27 accumulation by inhibition of proteasome function induces apoptosis in oral squamous cell carcinoma cells. Clin Cancer Res 2000 6 916 23 10741716

[33] Yoshida K Nakayama K Nagahama H Involvement of p27(KIP1) degradation by Skp2 in the regulation of proliferation in response to wounding of corneal epithelium. Invest Ophthalmol Vis Sci 2002 43 364 70 11818378

[34] Walker JL Wolff IM Zhang L Menko AS Activation of Src kinases signals induction of posterior capsule opacification. Invest Ophthalmol Vis Sci 2007 48 2214 23 17460282 10.1167/iovs.06-1059

[35] Shui Y-B Arbeit JM Johnson RS Beebe DC HIF-1: An age-dependent regulator of lens cell proliferation. Invest Ophthalmol Vis Sci 2008 49 4961 70 18586877 10.1167/iovs.08-2118 PMC2585414

[36] Mailankot M Smith D Howell S Wang B Jacobberger JW Stefan T Nagaraj RH Cell cycle arrest by kynurenine in lens epithelial cells. Invest Ophthalmol Vis Sci 2008 49 5466 75 18676626 10.1167/iovs.08-2374 PMC2610264

[37] Malecaze F Couderc B de Neuville S Serres B Mallet J Douin-Echinard V Manenti S Revah F Darbon JM Adenovirus-mediated suicide gene transduction: feasibility in lens epithelium and in prevention of posterior capsule opacification in rabbits. Hum Gene Ther 1999 10 2365 72 10515456 10.1089/10430349950017013

[38] Couderc BC de Neuville S Douin-Echinard V Serres B Manenti S Darbon JM Malecaze F Retrovirus-mediated transfer of a suicide gene into lens epithelialcellsin vitroand in an experimentalmodel ofposterior capsule opacification. Curr Eye Res 1999 19 472 8 10550788 10.1076/ceyr.19.6.472.5284

[39] Malecaze F Decha A Serre B Penary M Duboue M Berq D Levade T Lubsen NH Kremer EJ Couderc B Prevention of posterior capsule opacification by the induction of therapeutic apoptosis of residual lens cells. Gene Ther 2006 13 440 8 16251995 10.1038/sj.gt.3302667

[40] Kampmeier J Behrens A Wang Y Yee A Anderson WF Gordon EM Inhibition of rabbit keratocyte and human fetal lens epithelial cell proliferation by retrovirus-mediated transfer of antisense cyclin G1and antisense MAT1constructs. Hum Gene Ther 2000 11 1 8 10646634 10.1089/10430340050016102

